# The ribosomal protein gene RPL5 is a haploinsufficient tumor suppressor in multiple cancer types

**DOI:** 10.18632/oncotarget.14895

**Published:** 2017-01-29

**Authors:** Laura Fancello, Kim R. Kampen, Isabel J.F. Hofman, Jelle Verbeeck, Kim De Keersmaecker

**Affiliations:** ^1^ KU Leuven-University of Leuven, Department of Oncology, LKI-Leuven Cancer Institute, Leuven, Belgium

**Keywords:** ribosomal protein, haploinsufficient tumor suppressor, TCGA, breast cancer

## Abstract

For many years, defects in the ribosome have been associated to cancer. Recently, somatic mutations and deletions affecting ribosomal protein genes were identified in a few leukemias and solid tumor types. However, systematic analysis of all 81 known ribosomal protein genes across cancer types is lacking. We screened mutation and copy number data of respectively 4926 and 7322 samples from 16 cancer types and identified six altered genes (*RPL5*, *RPL11*, *RPL23A*, *RPS5*, *RPS20* and *RPSA*). *RPL5* was located at a significant peak of heterozygous deletion or mutated in 11% of glioblastoma, 28% of melanoma and 34% of breast cancer samples. Moreover, patients with low *RPL5* expression displayed worse overall survival in glioblastoma and in one breast cancer cohort. *RPL5* knockdown in breast cancer cell lines enhanced G2/M cell cycle progression and accelerated tumor progression in a xenograft mouse model. Interestingly, our data suggest that the tumor suppressor role of RPL5 is not only mediated by its known function as TP53 or c-MYC regulator. In conclusion, *RPL5* heterozygous inactivation occurs at high incidence (11-34%) in multiple tumor types, currently representing the most common somatic ribosomal protein defect in cancer, and we demonstrate a tumor suppressor role for RPL5 in breast cancer.

## INTRODUCTION

Cancers contain a variety of genomic lesions including mutations, translocations, copy number alterations and epigenetic changes that can result in altered protein functions. Several functional protein categories show somatic alterations in cancer, such as transcription factors, signaling molecules and epigenetic regulators. However, only recently somatic defects in ribosomal proteins have been described in tumors. The human ribosome corresponds to the cellular machinery translating mRNA into proteins and is composed of a small 40S subunit consisting of the 18S rRNA chain and 33 RPS proteins and a large 60S subunit encompassing the 28S, 5S and 5.8S rRNA chains and 47 RPL proteins. Somatic mutations and deletions affecting ribosomal protein genes occur in up to 20% of acute T-cell leukemia (T-ALL) cases, with the most frequent defects affecting *RPL10* (also known as uL16; 7.9% of pediatric T-ALL cases) and *RPL22* (eL22; 10%) and with rare defects in RPL5 (uL18) and RPL11 (uL5) [1–3]. Moreover, somatic mutations affecting RPS15 (uS19) were reported in 10-20% of aggressive chronic lymphocytic leukemia (CLL) [4, 5]. The Cancer Genome Atlas (TCGA) pan-cancer analyses identified *RPL5* and *RPL22* as significantly mutated in glioblastoma multiforme (GBM, 2.8%) and uterine corpus endometrial carcinoma (UCEC, 10.9%) respectively [6–9], and inactivating *RPL22* mutations have also been described in colorectal and gastric cancer [10, 11]. *RPS27* contains a mutational hotspot in its 5’UTR in melanoma (SKCM) [12], and we showed that *RPL5* is part of a minimal deleted region that is heterozygously deleted in 20-40% of advanced multiple myeloma cases [13].

Mutations reported for *RPL10* in T-ALL are all missense mutations, with a strong mutational hotspot at residue arginine 98 (R98S) [1], indicating an oncogenic role for these mutations. In contrast, all other somatic defects that have been identified so far in ribosomal protein genes are heterozygous and many of them are clearly inactivating mutations or deletions, suggesting roles as haploinsufficient tumor suppressors for these proteins in cancer [1–8].

Congenital heterozygous inactivating mutations and deletions affecting *RPL5*, *RPL11* and *RPS15* have also been described in Diamond Blackfan Anemia (DBA), a congenital syndrome belonging to a family of human disorders, ribosomopathies, caused by impaired ribosome biogenesis and function [14, 15]. Similarly, *RPS14 (uS11)* haploinsufficiency has been reported in the 5q- myelodysplastic syndrome (MDS) [16]. Like other ribosomopathies, DBA and 5q- MDS patients are characterized by hypoproliferative phenotypes such as bone marrow failure and anemia early in life, followed by elevated cancer risks later on [14, 15, 17].

Besides the identification of somatic ribosome defects in cancer and the elevated cancer risks of ribosomopathy patients, the link between ribosome defects and cancer is supported by the observation that heterozygous inactivation of certain ribosomal protein genes induces tumor development in zebrafish [18]. Moreover, *Rpl11* and *Rpl22* haploinsufficiency accelerates mouse lymphoma development and loss of one copy of *Rpl5 or Rps24 (eS24)* has been linked to development of rare soft tissue sarcomas in mice [2, 19, 20]. Some of the ribosomal proteins affected in cancer have also been linked to known prominent oncogenes and tumor suppressors. In this context, several ribosomal proteins have been reported to bind and sequester MDM2 and activate the p53 pathway upon ribosomal stress, although only RPL5, RPL11 and RPL23 are essential for this process [21–27]. Additionally, certain ribosomal proteins regulate c-MYC, which itself is responsible for ribosome biogenesis by stimulating transcription of ribosomal RNA and proteins [28–30]. In this context, RPL5, RPL11 and RPS14 suppress c-MYC expression by guiding the c-*MYC* mRNA to the RISC complex for degradation [31–33]. Moreover, RPL11 interacts with c-MYC at promoter regions of c-MYC target genes, inhibiting its transcriptional activity [34, 35]. *RPL22* inactivation was reported to indirectly activate c-*MYC* expression, via an NF-kB - Lin28B - Let7 miRNA axis [2].

So far, a small set of ribosomal protein genes has been found mutated in cancer. However, systematic analysis of all 81 ribosomal protein genes across cancer types is lacking, and certain defects may have been overlooked. In this study, the mutation and copy number data within the TCGA database were explored to provide the first systematic screening of potential cancer driving ribosomal protein genes, which may reveal interesting novel targets to investigate in follow-up studies. We analyzed mutations from 4 926 tumors and copy number changes from 7 322 tumors across 16 different tumor types for the 81 genes encoding ribosomal proteins. Six ribosomal protein genes were identified as potential cancer drivers in five different cancer types. Among them, *RPL5* was the strongest candidate affected in 11-34% of glioblastoma, melanoma and breast cancer patients. Whereas somatic mutations in *RPL5* had been described in 3% of GBM samples, we found that *RPL5* heterozygous inactivation currently represents the most common somatic ribosome defect in human cancer. Importantly, 50% reduction of RPL5 levels in breast cancer cell lines increased cell proliferation and tumor progression in mouse xenograft models, further supporting a haploinsufficient tumor suppressor role for RPL5 in human cancer.

## RESULTS

### TCGA screening identifies 6 ribosomal protein genes with a candidate driver role in cancer

**Figure 1:**
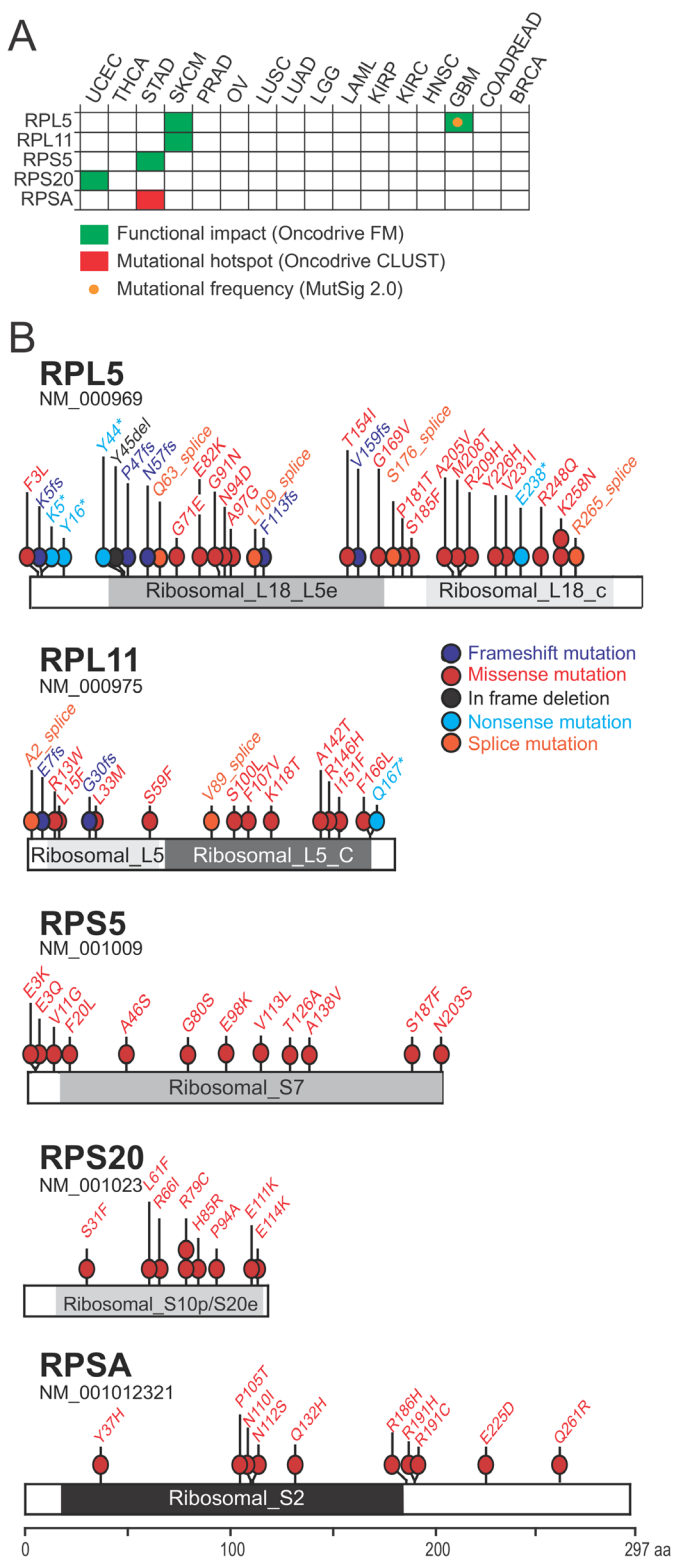
Identification of 5 ribosomal protein genes that are significantly mutated in cancer (**A**) Significantly mutated genes identified due to mutational frequency (MutSig 2.0), mutational clustering (OncodriveCLUST) or accumulation of high functional impact mutations (OncodriveFM). (**B**) Mapping of mutations affecting the 5 candidate cancer drivers on linear protein diagrams. Non-silent somatic mutations from all 16 cancer types are shown. Protein domains are indicated for each protein. Ribosomal_L18_L5e (pfam00861), Ribosomal_L18_c (pfam14204): RPL5 protein domains. Ribosomal_L5 (pfam00281), Ribosomal_L5_C (pfam00673): RPL11 protein domains. Ribosomal_S7 (pfam00177): RPS5 protein domain. Ribosomal_S10p/S20e (pfam00338): RPS20 protein domain. Ribosomal_S2 (pfam00318): RPSA protein domain.

The TCGA database was explored to identify ribosomal protein genes that are significantly altered in cancer. The first type of alteration that was analyzed were non-silent somatic mutations. Overall, the frequency of such defects in individual ribosomal protein genes was below 3% in all cancer types (Supplementary Table 2). However, mutational frequency represents only one criterium to discriminate functional cancer drivers. Clustering of mutations in a particular protein region also indicates positive selection, as well as accumulation of mutations with high impact on protein function. While some cancer genes are mutated at high frequency (e.g. *TP53* or *KRAS*), most cancer genes are mutated at much lower frequencies (2-20%) [8]. Therefore, we retained all genes with significant mutational frequency (as determined by MutSig 2.0) and/or positional clustering of mutations (OncodriveCLUST) and/or accumulation of high functional impact mutations (OncodriveFM). According to these criteria, five genes (*RPL5*, *RPL11*, *RPS5 (uS7), RPS20 (uS10), RPSA (uS2)*) were significantly mutated in four different cancer types (Figure 1A-1B).

*RPSA* was identified in Stomach Adenocarcinoma (STAD) because of a significant cluster of mutations (q-value: 0.004; OncodriveCLUST ) (Figure 1A-1B; Supplementary Figure 1). *RPS5* and *RPS20* show an accumulation of high functional impact mutations, in STAD and UCEC respectively (q-values: 0.026 and 0.042; Oncodrive FM). Interestingly, a few of these mutations are predicted to affect the interaction between the ribosomal protein and RNA (Supplementary Table 3). Accumulation of high functional impact mutations was also found for *RPL5* in GBM (q-value: 0.0002) and SKCM (q-value: 0.004) and for *RPL11* in SKCM (q-value: 0.0007). Some of these mutations were clearly inactivating frameshift, nonsense or splice site mutations (Figure 1B), indicative of a tumor suppressor function. Finally, *RPL5* was the only gene with a significantly high mutational frequency according to MutSig 2.0 and the only gene significantly mutated in 2 different cancer types.

**Figure 2:**
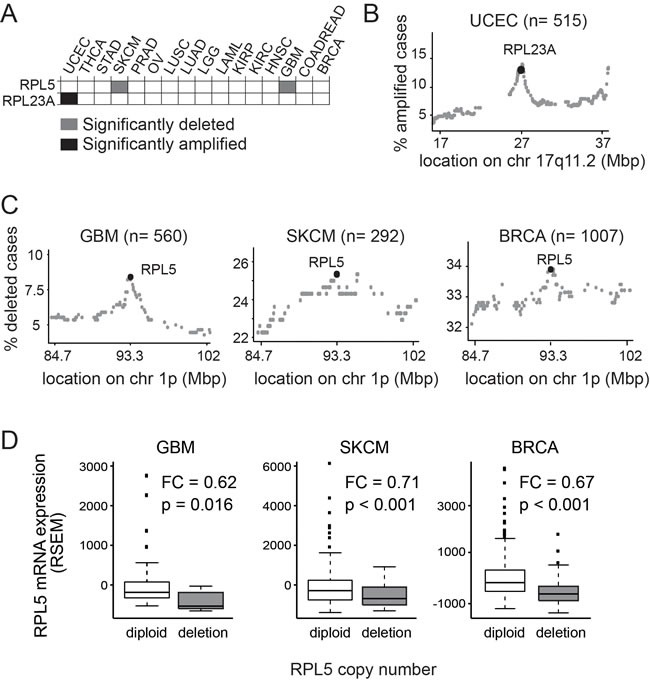
RPL5 and RPL23A show significant copy number changes in the TCGA database (**A**) Heatmap showing the significant copy number changes retained (**B**) *RPL23A* amplification peak in UCEC. Each dot on the figure represents a different gene on chr 17q11.2, the genomic locus where the *RPL23A* gene is located. X-axis: genomic coordinates on chromosome 17; Y-axis: percent of cases with amplification of each particular gene. (**C**) *RPL5* deletion peak in GBM, SKCM and BRCA. Each dot on the figure represents a different gene on chr 1p, the genomic locus where the *RPL5* gene is located. X-axis: genomic coordinates on chromosome 1; Y-axis: percent of cases with deletion of each particular gene. (**D**) Boxplots showing *RPL5* mRNA expression levels (RSEM) in GBM, SKCM and BRCA cases with diploid or heterozygously deleted copy number status for *RPL5*. P: p-value according to the Wilcoxon's test. FC: fold change (*RPL5* heterozygously deleted over *RPL5* diploid).

We also screened the TCGA database for ribosomal protein genes affected by significant copy number changes (Supplementary Figure 2). Because these defects often encompass many genes, we increased the specificity of our screening for driver events by applying additional filtering criteria: i) that the ribosomal protein gene was included in the region of the deletion (or amplification) that is predicted to contain the cancer driving target gene; ii) that the same region does not include other known cancer genes; iii) that the ribosomal protein gene was also affected by mutations, in addition to the significant copy number change. Only 2 genes, *RPL23A (uL23)* and *RPL5*, were retained after this filtering (Figure 2A).

*RPL23A* was significantly amplified in UCEC (Figure 2A). Interestingly, *RPL23A* was located in a distinct peak of amplification encompassing only 24 genes and was amplified in 12.62% (*n* = 65) of UCEC samples (Figure 2B). Patient samples harboring *RPL23A* amplification displayed 1.5 fold higher average *RPL23A* mRNA expression levels compared to *RPL23A* diploid tumor samples (Wilcoxon test, W = 3316, *p* = 0.003) (Supplementary Figure 3A). No evidence was found for effects on patients’ survival but *RPL23A* amplified cases were more frequent among the serous endometroid tumors, a more rare and aggressive UCEC subtype (q-value: 2.08E-018) (Supplementary Figure 3B).

*RPL5* was heterozygously deleted in 8.4% of GBM and 25.3% of SKCM patients and in both cancers the *RPL5* gene locus was positioned in a distinct focal peak of deletion (Figure 2C). Furthermore, in breast invasive carcinoma (BRCA), *RPL5* was significantly heterozygously deleted in 33.9% of cases. These defects were initially not retained in our filtering, because known cancer genes (*NRAS*, *BCL10*, *TRIM33*, *RBM15*) are also included in the same deletion and the minimal mutational frequency requirement was not satisfied. However, a closer analysis showed that the known cancer genes are more than 7 Mbp away from *RPL5* and also in this tumor type *RPL5* was located in a pronounced deletion peak (Figure 2C). *RPL5* deletions were associated with 29-38% lower average *RPL5* mRNA expression levels in GBM (*p* = 0.016), SKCM (*p* = 2.36e-04) and BRCA (*p* = 2.2e-16) (Figure 2D).

In conclusion, we defined 6 ribosomal protein genes as candidate cancer driving genes (Table 1). *RPL5*, the most commonly altered ribosomal protein we detected, was significantly mutated and deleted in GBM (11%) and SKCM (28%) and significantly deleted in BRCA (34%).

**Table 1:** Aberrations in six candidate driver genes

Gene	Cancer	Aberration	# Analyzed Tumors	% Tumors with Aberration
RPL5	SKCM	mutations	279	2.5
		deletions	293	25.3
	GBM	mutations	283	2.5
		deletions	560	8.4
	BRCA	mutations	976	0.2
		deletions	1016	33.9
RPL11	SKCM	mutations	279	1.4
RPL23A	UCEC	mutations	248	2.0
		amplifications	515	12.6
RPS5	STAD	mutations	221	1.4
RPS20	UCEC	mutations	248	1.2
RPSA	STAD	mutations	221	2.7

### *RPL5* is a clinically relevant candidate tumor suppressor in GBM

**Figure 3:**
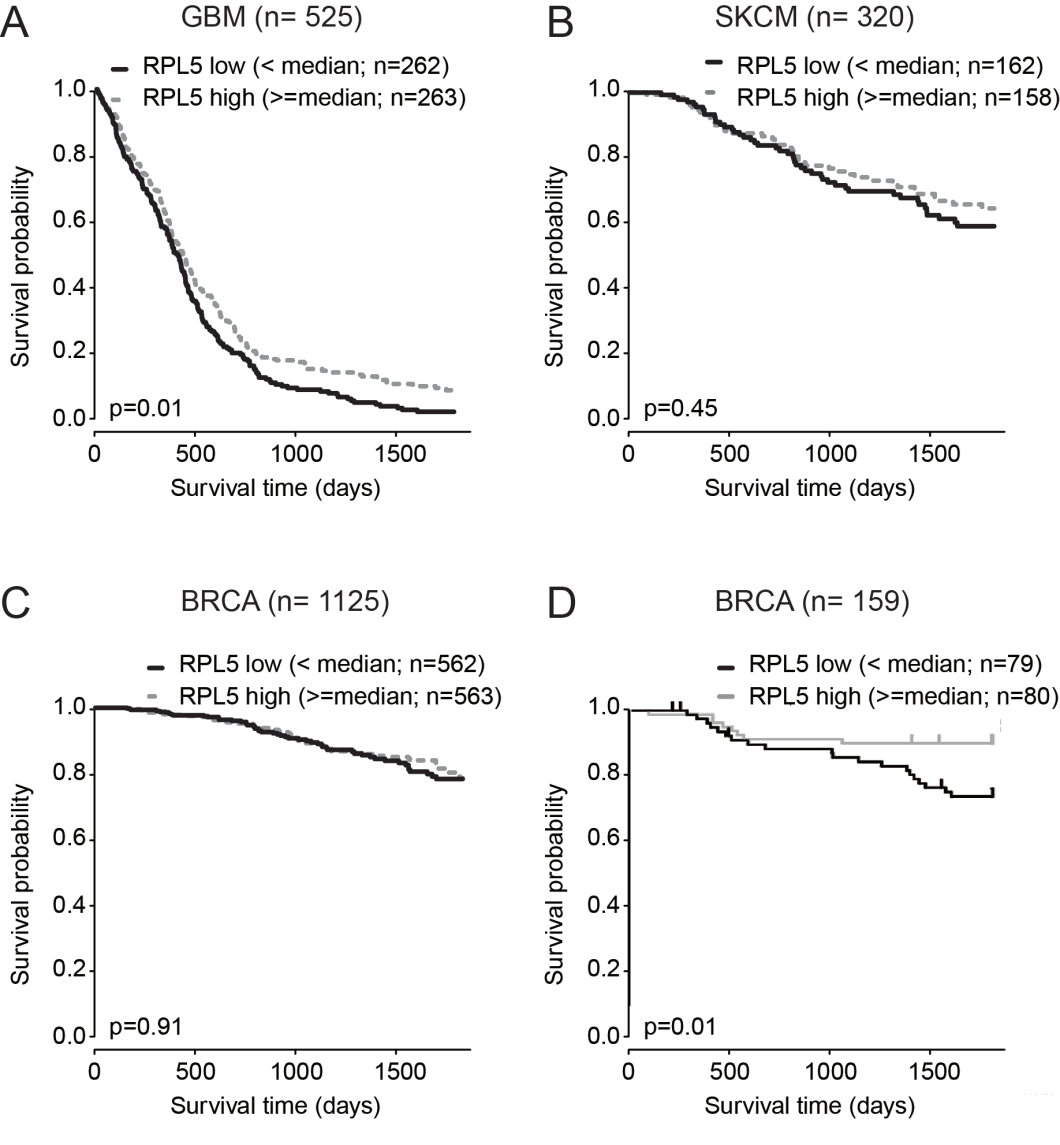
RPL5 is a clinically relevant candidate tumor suppressor in GBM Kaplan–Meier analysis of the effect of *RPL5* expression on overall survival. Cases were divided in RPL5 low or high expressers according to whether expression was below or above median and survival was compared using the log-rank test. (**A**) GBM TCGA dataset; (**B**) SKCM TCGA dataset; (**C**) BRCA TCGA dataset; (**D**) Non-TCGA BRCA dataset available on the R2 platform (GEO accession: GSE1456).

For each of the 6 identified candidate cancer drivers, we evaluated the impact of expression levels on overall survival. Only for *RPL5,* a significant association was found. In GBM, low *RPL5* expression was associated with a reduced five-year overall survival (p = 0.01) (Figure 3A). The median survival for patients with low *RPL5* expression (*n* = 414) was 13.8 months, whereas this was 14.7 months for high *RPL5* expressing patients (*n* = 442). For BRCA or SKCM no significant difference in survival was found in the TCGA datasets (Figure 3B-3C). However, an additional non-TCGA BRCA dataset from the Pawitan *et al*. study (GEO accession: GSE1456) available in the R2 platform was analyzed. In that dataset, a significant correlation of *RPL5* low expression with worse five year survival was found (*p* = 0.01) (Figure 3D). Unfortunately, R2 did not contain additional datasets suitable for our survival analyses for GBM or SKCM.

### RPL5 defects co-occur with TP53 pathway inactivation and c-MYC amplification in SKCM and BRCA

**Figure 4:**
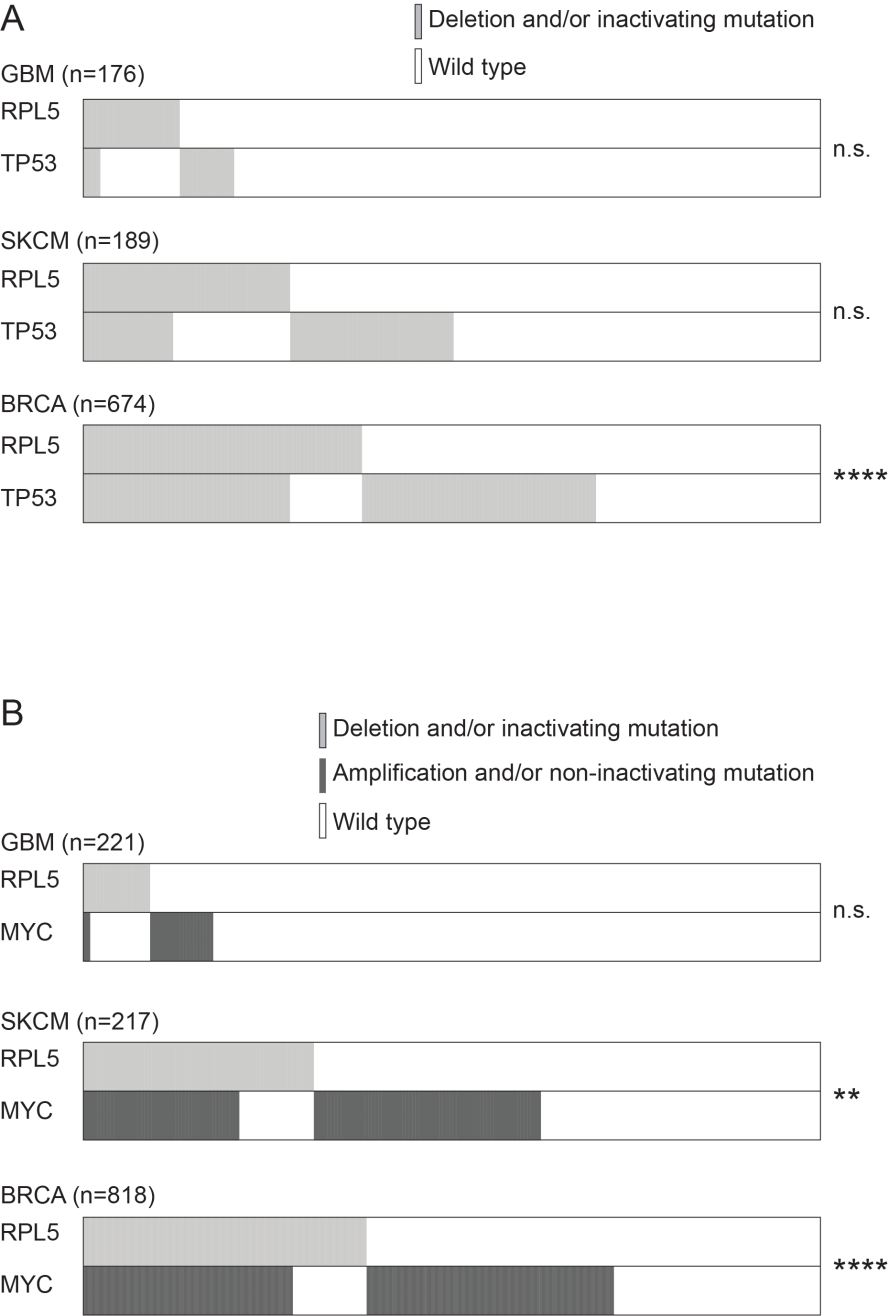
Genetic interaction between RPL5 alterations and TP53 or c-MYC alterations (**A**) Co-occurrence of *RPL5* and *TP53* alterations in GBM, SKCM and BRCA. (**B**) Co-occurrence of *RPL5* and *c-MYC* alterations in GBM, SKCM and BRCA.

Because RPL5 has been functionally linked to TP53 and c-MYC, we tested for an association between *RPL5* defects and defects in *c-MYC*, *TP53*, or the negative TP53 regulators *MDM4* and *MDM2* (Figure 4 and Supplementary Figure 4). In BRCA, significant co-occurrence of *RPL5* defects and *TP53* pathway inactivation by *TP53* inactivation or by mutation/amplification of *MDM2* or *MDM4* was detected. In this cancer type, also *c-MYC* amplification co-occurred with *RPL5* inactivation. Also in SKCM, a significant co-occurrence of *RPL5* defects with *MDM4*, *MDM2* and *c-MYC* amplification was detected. No significant associations were obtained in GBM, which might be due to lower sample numbers from which data were available.

Furthermore, we assessed the mRNA levels of the TP53 target genes CDKN1A, BAX and MDM2, as well as the mRNA levels of c-MYC. No significant changes were found between *RPL5* diploid and *RPL5* heterozygously deleted tumors (Supplementary Figure 5).

### RPL5 knockdown enhances breast cancer cell proliferation and accelerates tumor formation in mice

**Figure 5:**
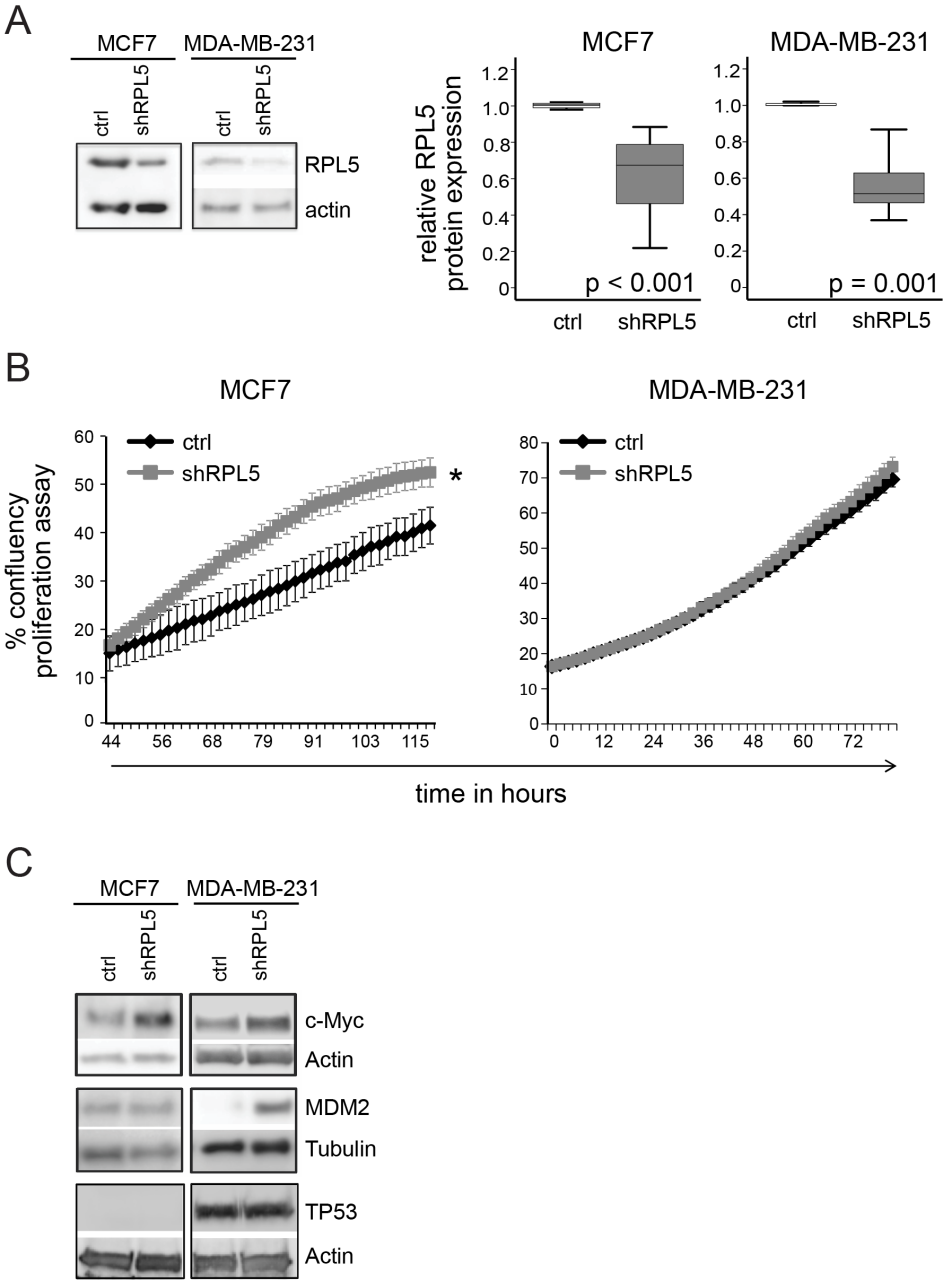
RPL5 knockdown enhances proliferation of MCF7 breast cancer cells (**A**) Immunoblot analysis of RPL5 expression levels on cell lysates of doxycycline treated MCF7 and MDA-MB-231 cell lines containing an empty lentiviral vector (control) or a vector containing an shRNA targeting RPL5 (shRPL5). Quantification of the blots is shown on the right. (**B**) *In vitro* proliferation of MCF7 and MDA-MB-231 cell lines as determined by real-time monitoring of cell confluency. (**C**) Immunoblot analysis of c-MYC, TP53 and MDM2 expression levels on cell lysates of doxycycline treated MCF7 and MDA-MB-231 cell lines containing an empty lentiviral vector (control) or a vector containing an shRNA targeting RPL5 (shRPL5). All immunoblots were performed 72 hrs after start of the doxycycline treatment.

**Figure 6:**
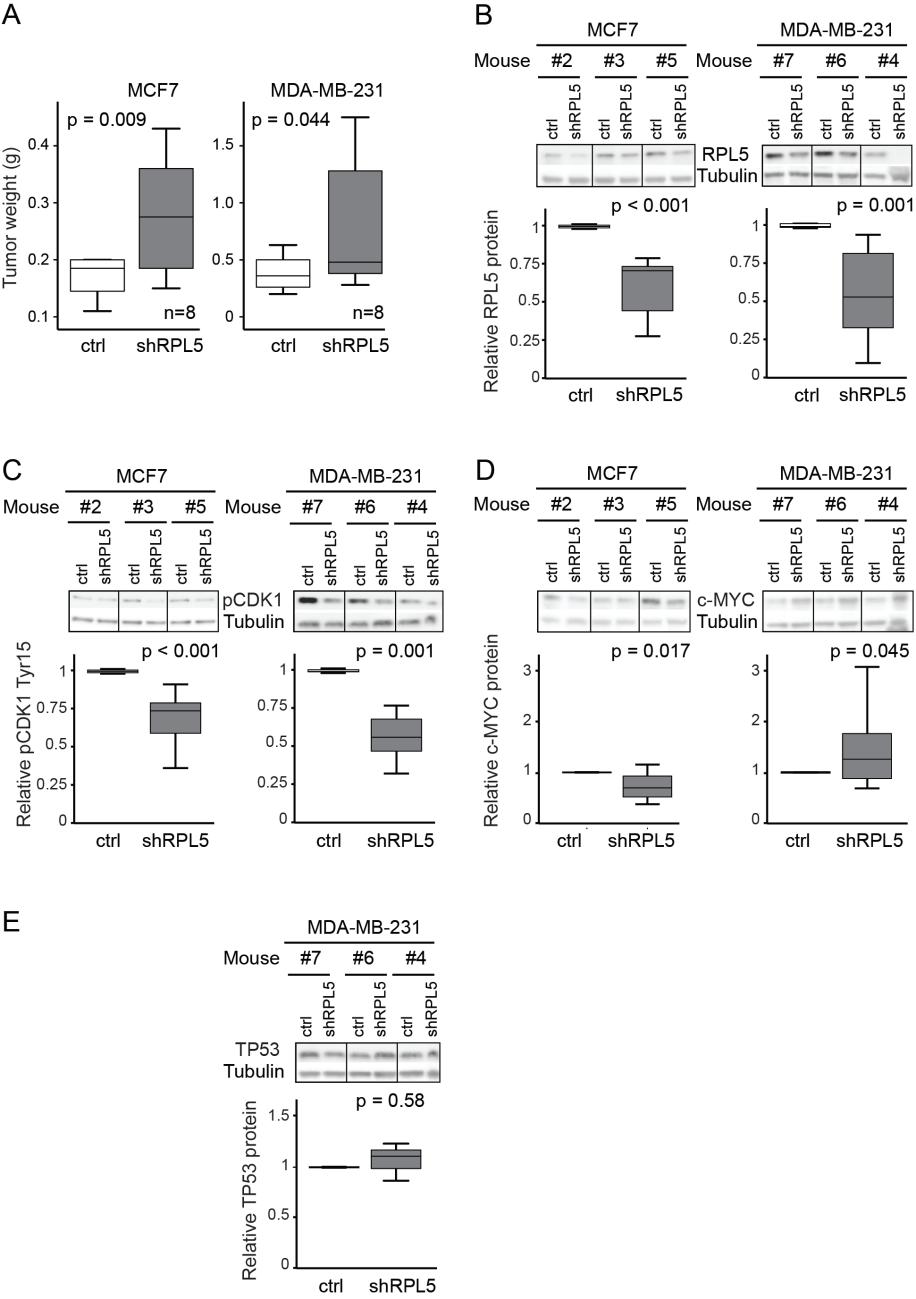
Knockdown of RPL5 accelerates breast cancer formation in mice (**A**) Tumor weights of doxycycline treated mice that are injected with MCF7 and MDA-MB-231 cell lines containing the empty lentiviral vector (control) in the left flanks and the vector containing an shRNA targeting RPL5 (shRPL5) in the rights flanks. (**B**-**E**) Immunoblot analysis and the corresponding quantification of MCF7 (left) and MDA-MB-231 (right) tumors, comparing expression of the control and shRPL5 condition for RPL5 (**B**), phospho-CDK1 (tyr15) (**C**), c-MYC (**D**), and TP53 protein (**E**).

*RPL5* emerged as the strongest candidate cancer driver gene from our analysis. It represents a candidate tumor suppressor, based on its heterozygous inactivating mutations and focal deletions and based on the correlation of lower *RPL5* expression with worse survival. Therefore, we aimed to experimentally test the effect of ~50% *RPL5* loss-of-function on cell behavior *in vitro* and on tumor forming capacity in an *in vivo* mouse model. We chose for breast cancer models, because of the high incidence of *RPL5* inactivation in BRCA. Triple negative breast cancer cell lines MCF7 (*TP53* WT, *RPL5* WT) and MDA-MB-231 (*TP53* homozygous R280K missense mutation, *RPL5* WT) were transduced with lentiviral vectors allowing inducible *RPL5* protein knockdown of 30-50% (Figure 5A, Supplementary Figure 6, MCF7 *p* < 0.001 and MDA-MB-231 *p* = 0.001). Interestingly, this RPL5 knockdown induced proliferation of MCF7 cells, but not of MDA-MB-231 cells (Figure 5B, MCF7 *p* < 0.001 and MDA-MB-231 *p* = 0.597). Next, we injected the breast cancer cell lines subcutaneously into the left (ctrl vector) and right (sh*RPL5* vector) flanks of NSG mice to identify the role of *RPL5* in breast cancer progression. MDA-MB-231 tumors grew faster (one month) as compared to MCF7 tumors (two months). In both subcutaneous breast cancer models, *RPL5* knockdown significantly increased the tumor weight when sacrificing the animals (Figure 6A, MCF7 *p* = 0.009 and MDA-MB-231 *p* = 0.044). It was noticed that in 2/8 (25%) of the mice with MDA-MB-231 induced tumors, the right hind leg was fixated and the animal was not using it anymore. After dissection, it was clear that in these mice, the sh*RPL5* tumors had encapsulated the bone (Supplementary Figure 7). This was not observed in the more slowly growing MCF7 tumors. All tumors were analyzed by immunoblot and showed clear knockdown of *RPL5* (Figure 6B, MCF7 *p* < 0.001 and MDA-MB-231 *p* = 0.001). In addition, the sh*RPL5* induced mouse tumors showed reduced phosphorylation of CDK1/CDC2 at tyrosine 15, a dephosphoryation that is required for cell cycle progression from G2 to mitosis (Figure 6C, MCF7 *p* < 0.001 and MDA-MB-231 *p* = 0.001). These results are consistent with the enhanced proliferation associated with *RPL5* knockdown in the cell culture experiments and the increased tumor weights *in vivo*.

Changes in TP53, MDM2 and c-MYC protein levels upon *RPL5* knockdown were assessed by immunoblotting, *in vitro* and *in vivo* (Figure 5C, Figure 6D-6E and Supplementary Figure 8). *TP53* protein could not be detected for the MCF7 cell line or tumors and no differences were observed for MDA-MB-231. MDM2 expression did not differ *in vivo* and it was upregulated *in vitro* only for MDA-MB-231. Expression of *c-MYC* was significantly upregulated in both cell lines *in vitro* and in MDA-MB-231 tumors (*p* = 0.045) but downregulated in MCF7 tumors (*p* = 0.017).

## DISCUSSION

Using the TCGA database, we performed the first screening exploring the incidence and pattern of somatic defects affecting the 81 ribosomal protein genes in cancer. We aimed to identify novel ribosomal protein genes which are significantly targeted by genetic alterations and represent potential causative cancer genes. Our screening for mutations and copy number changes identified six ribosomal protein genes as candidate cancer driver genes, including *RPL5* and *RPL11*, which were previously reported in cancer. Our analyses did not pick up *RPL22*, *RPS27 (eS27*)*, RPS15* and *RPL10*, although already described in cancer as well. *RPL22* was found to be significantly mutated in UCEC, according to a TCGA pan-cancer analysis [6, 7]. However, most *RPL22* mutations detected in TCGA UCEC are potentially caused by misalignment of reads to homologous regions in the genome. Therefore, these mutations were absent in the more recent Firehose release of TCGA data used in our study. *RPS27* has been reported to contain a mutational hotspot in its 5’UTR in SKCM [12]. This was not detected here because our analyses were restricted to the protein coding regions and splice sites of ribosomal protein genes. *RPS15* and *RPL10* mutations have been reported in CLL and T-ALL [1, 4, 5], which are not represented in the TCGA dataset analyzed here. However, our data suggest that these ribosomal protein genes are not affected in cancer in general and that these mutations may be specific to the disease entities where they have been described. This specificity may relate to the emerging idea of “specialized ribosomes”, according to which ribosomes present a heterogeneous composition and/or binding capacity to translation factors in different cell types [36, 37]. This may alter their functionality and contribute to tissue specific gene regulation. Alternatively, cell type specific extra-ribosomal roles for these ribosomal proteins may exist.

The reasons mentioned above explain the absence of previously reported cancer associated ribosomal protein defects in our results. In addition, we only considered mutations and copy number changes in ribosomal protein genes. Other mechanisms such as methylation, regulation by microRNAs or long non-coding RNAs might further cause ribosomal protein dysregulation in cancer. Because of all these reasons, we do not claim to have fully delineated the spectrum of ribosomal protein defects in cancer here, but we identified 6 attractive genes for further investigation.

Among the candidate genes detected in this screening, *RPSA* harbors a mutational hotspot likely to influence its extra-ribosomal function as laminin binding protein. Besides being a constituent of the ribosome, RPSA also functions on the extracellular membrane as a laminin receptor and transduces extracellular signals regulating cancer-related pathways such as apoptosis and cell migration [38, 39]. The mutational hotspot we detected was located in a flexible loop whose conformation regulates the accessibility of a laminin binding domain. Additionally, two mutations were close to the cleavage sites which regulate laminin-binding (Supplementary Figure 1). Both the laminin-binding protein RPSA and the metalloproteinase ST3 responsible for its cleavage are known to be overexpressed in different cancers [40–42]. Our data may indicate a role for RPSA in oncogenesis through its extra-ribosomal function as laminin receptor.

*RPL23A* amplification in UCEC patients may represent a prognostic factor for a more aggressive and rare UCEC histological type. *RPS5* and *RPS20* show accumulation of high functional impact mutations, some of which may disrupt RNA interactions. Most interestingly, both *RPL11* and *RPL5* present inactivating mutations and emerge as candidate cancer drivers in the same cancer type (SKCM). These two ribosomal proteins are part of the same complex regulating TP53 via MDM2 and are both mutated and deleted in patients affected by DBA, a cancer predisposing ribosomopathy [14, 21, 23, 27, 43–45]. No other ribosomal protein genes associated to DBA or other ribosomopathies are detected among the six candidate cancer driver genes identified in this study.

*RPL5* was the strongest candidate cancer gene emerging from our screening. The observation of heterozygous inactivating *RPL5* mutations and deletions across multiple tumor types suggested a role as haploinsufficient tumor suppressor gene. It is interesting to note that the Rabadan group integrated TCGA data with known causative genes in cancer predisposing Mendelian diseases, such as DBA, and that they also pick up RPL5 as candidate tumor suppressor in GBM in their analyses [46]. *RPL5* has previously been identified as significantly mutated in GBM (2.8%) [6–8]. However, the incidence of *RPL5* alterations was severely underestimated in these studies, since no copy number changes were considered. In the current study, we show that the incidence of *RPL5* alterations in GBM is much higher than previously assumed and that heterozygous *RPL5* inactivation occurs at high incidence in GBM, SKCM and BRCA. As such, *RPL5* inactivation currently represents the most common somatic ribosome defect in cancer. It is worth pointing out that *RPL5* defects in other tumor types did not pass the threshold for retention in our pipeline. In this context, LUAD, KIRC, STAD and PRAD also show inactivating mutations or deletions in *RPL5* (Supplementary Figure 9), suggesting that *RPL5* may also act as a tumor suppressor in these tumor types. In addition, *RPL5* shows inactivating mutations in 2% of T-ALL samples and we showed that *RPL5* is part of a minimal deleted region that is heterozygously deleted in 20-40% of advanced multiple myeloma cases (Supplementary Figure 10) [1, 13].

We experimentally validated the role of RPL5 as haploinsufficient tumor suppressor in breast cancer cell and mouse models. Knockdown of *RPL5* by ~50% in both TP53 WT MCF7 and *TP53* mutant MDA-MB-231 breast cancer lines accelerated the tumor growth *in vivo*. In agreement with this, *RPL5* knockdown tumors showed reduced phosphorylation of *CDK1/CDC2* at tyrosine 15, which requires dephosphorylation to enable cell cycle progression through the G2/M checkpoint. The MDA-MB-231 cell line did not show significant proliferation changes *in vitro* upon *RPL5* knockdown, although a significant increase in tumor growth was observed *in vivo*. It is well known that the *in vivo* setting is an important factor in tumor progression. Growth factor release by a supportive tumor microenvironment or other mechanisms such as tumor hypoxia, which cannot be mimicked *in vitro*, may explain why the *RPL5* knockdown in MDA-MB-231 cells showed only a significant effect in the *in vivo* context.

We describe the first models for heterozygous *RPL5* inactivation in cancer cell context. *RPL5* haploinsufficiency systems have however previously been generated to study the effect of *RPL5* defects in the context of DBA. Loss of *RPL5* in human primary lung fibroblasts does not induce cell cycle arrest by checkpoint activation but suppresses cell cycle progression by reducing translation rates, including translation of some cyclins [22]. Haploinsufficiency of *RPL5* in mouse embryonic stem cell cultures causes growth defects with a delay in the G2/M cell cycle checkpoint [47]. It is speculated that differences in cell cycle checkpoint activation may be due to the distinct differentiation status of the cells used in these studies. However, both studies showed impeded proliferation upon *RPL5* loss, whereas we observed increased proliferation and tumor growth in our *RPL5* haploinsuffiency cancer model. The cancer context we adopted in our study likely explains this discrepancy by the required support of additional mutations. Interestingly, these opposite proliferation effects of *RPL5* haploinsufficiency recall the paradox of phenotypes associated to ribosomopathies. Patients affected by these diseases initially present a hypoproliferative phenotype (such as anemia) frequently followed, in later stages of the disease, by the development of cancer, a hyperproliferative disease [17]. The mechanisms underlying the initial hypoproliferation or the subsequent cancer development still have to be elucidated.

Our analysis of TCGA expression data did not reveal activation of TP53 target genes in BRCA, GBM or SKCM tumors with *RPL5* haploinsufficency, consistent with previous data in a non-cancer context [22, 47]. Moreover, we observed increased *in vivo* tumor volumes upon RPL5 knockdown, both in the TP53 wild type MCF7 and in the TP53 mutant MDA-MB-231 line and no consistent changes were observed in TP53 and MDM2 protein expression upon knock-down of RPL5 in these breast cancer cell lines. These results suggest that the *RPL5* knockdown phenotype is likely *TP53*-independent. Alternatively, since *RPL5* and *RPL11* are known to cooperate in suppressing expression of c-*MYC*, *RPL5* loss may induce upregulation of this potent oncogene [31, 32]. No differences in c-*MYC* mRNA levels was found for BRCA, GBM or SKCM tumors with *RPL5* haploinsufficency from TCGA. c-MYC protein was upregulated in both breast cancer cell lines with *RPL5* knockdown *in vitro* and in MDA-MB-231 tumors *in vivo* but downregulated in MCF7 tumors. c-MYC overexpression in MDA-MB-231 cells has previously been shown to increase tumor volumes *in vivo* [48] and may have contributed to faster tumor growth as compared to MCF7 tumors. However, c-MYC upregulation does not fully explain the proliferation and tumor growth advantage conferred by *RPL5* knockdown since it also occurs in MCF7 tumors despite c-MYC downregulation. In summary, our data indicate that previously described extra-ribosomal functions of RPL5 regulating cancer genes *TP53* and c-*MYC* cannot explain the observed phenotype, although c-MYC regulation may partially contribute to it. Interestingly, our analysis of TCGA patient data from BRCA showed significant co-occurrence between *RPL5* and *TP53* or its negative regulator *MDM4* alterations. Similarly, a significant co-occurrence between *RPL5* and c-*MYC* alterations was identified. These results may suggest that alterations targeting the *TP53* pathway or c-*MYC* may co-operate with *RPL5* in tumorigenesis, and/or that *RPL5* inactivation may facilitate acquisition of these lesions.

In summary, we provide the first comprehensive analysis of defects in coding regions of ribosomal proteins across several cancer types using the TCGA platform. We identify RPSA, RPS5, RPS20, RPL5, RPL11 and RPL23A as six interesting cancer driver candidates and show a tumor suppressor role for RPL5 in the context of breast cancer. Additional research is required to experimentally evaluate the contribution of the other identified ribosomal protein defects in cancer pathogenesis. Similarly, more studies are needed to unravel the molecular mechanisms by which RPL5 exerts its tumor suppressor role in cancer.

## MATERIALS AND METHODS

### Data description

Data from TCGA (http://cancergenome.nih.gov/) and pre-processed by the Broad GDAC Firehose pipeline were used in this study (Firehose release of 15/01/2014). Only cancer types for which mutation and copy number data from at least 100 patients were available were included (Supplementary Table 1).

### Mutation analysis

Somatic mutation frequencies were obtained from the MAF files available on Firehose. Only somatic, non-silent mutations were considered. A gene was considered as significantly mutated in a cancer type if presenting a significantly high frequency of mutations (MutSig2.0) [49], or a significant mutational clustering in a particular region (OncodriveCLUST) [50], or a significant accumulation of mutations with predicted high impact on protein function (OncodriveFM) [51]. MutSig2.0 analyses were retrieved from Firehose. OncodriveCLUST and OncodriveFM analyses were generated on the IntOGen Mutations 2.4.1 platform [52].

Visualization of mutated residues on the protein 3D structure was generated in MuPIT interactive [53]. Mechismo was used to predict the functional impact of residue changes on protein and RNA interactions and TransFIC was used to estimate the functional impact of mutations [54, 55].

### Copy number analyses

Copy number values were retrieved from Firehose. These copy number values were estimated from Affymetrix SNP6.0 arrays as part of the Gistic 2.0 pipeline [56]. For a gene to be considered significantly deleted (or amplified) in a tumor entity, the following criteria had to be met: i) presence in a significant peak of deletion (or amplification) according to Gistic2.0; ii) presence in the ‘wide peak’ region predicted by Gistic2.0 that most likely harbors the target genes of the deletion (or amplification); iii) absence of other known cancer genes from Cancer Gene Census in the same ‘wide peak’; iv) presence of mutations in at least 2% of samples; v) at least 5 samples in the tumor entity in which the gene is deleted (or amplified).

### Survival analysis

Kaplan-Meier curves were generated using the *survfit* function of the survival R package and genes associated with survival were identified using the log-rank test. To analyze the association between gene expression (stratified by median) and survival, RNAseq expression values were used in all cancer types, except in GBM, for which more microarray based expression data were available. Expression data were RMA (Robust Multi-Array Average) normalized counts from Affymetrix HG-U133A microarray platform for GBM and Illumina HiSeq RSEM normalized counts for SKCM and BRCA.

The R2: Genomics Analysis and Visualization platform (http://r2.amc.nl) was used to generate Kaplan-Meier curves for *RPL5* expression in another BRCA dataset not included in TCGA (“Tumor Breast - Bergh - 159 - MAS5.0 - u133a”, GEO accession ID: GSE1456 [57]. Probe 213080_x_at was chosen and the median was used as cut-off for gene expression.

### Co-occurring and mutually exclusive mutations

The Fisher's exact test was used to identify significant co-occurrence or mutual exclusivity of genetic alterations in ribosomal protein genes and other genes. The interaction between *RPL5* inactivating mutations and deletions and *TP53* inactivating mutations and deletions and with *MDM2*, *MDM4* and c-*MYC* non inactivating mutations and amplifications was tested in BRCA, GBM and SKCM.

### Cell culture

MDA-MB-231 cells were obtained directly from ATCC. MCF7 cells originated from ATCC and were re-authenticated for this project by Microsynth AG. MCF7 and MDA-MB-231 were cultured in respectively RPMI-1640 and DMEM medium (Life Technologies) supplemented with 10% fetal bovine serum. Hek293T cells originate from DSMZ and were maintained in RPMI-1640. For proliferation assays, 12.500 cells/well were plated in a TPP 96-well plate. Cell proliferation was assessed by taking 4 pictures per well at 2 hour intervals and performing analysis of confluency on an IncuCyte Zoom system (Essen Bioscience). Each experiment was performed in three biological replicates and with a minimum of 6 technical replicates each time.

### Generation of *RPL5* knockdown cell lines

The doxycycline inducible LT3REVIR (pRRL) vector was a gift from Prof. Johannes Zuber (IMP, Vienna). shRNA sequence AGGAAATAGTGTGAAATTACAA targeting human *RPL5* was cloned into this vector and the resulting plasmid was transfected (Genejuice, EMD Millipore) into Hek293T cells to produce lentiviral supernatant using a VSV-G envelope and psPAX2 packaging plasmid. Cell medium was replaced 24 hrs after transfection, followed by viral supernatant collection after another 24h and transduction of the breast cancer cells (empty vector and *RPL5* shRNA) in the presence of 8 µg/mL polybrene (Sigma Aldrich). Cells were checked for transduction efficiency on a MACS VYB flow cytometer (Miltenyi), sorted (S3e, Biorad) and analyzed to check if the vector was switched on by 2 µg/mL doxycycline (Sigma Aldrich). Data were processed using FlowJo software.

### Immunoblotting

3*10^6^ cells or tumor tissue was lysed in cell lysis buffer (Cell Signaling Technologies). Protein concentrations were determined using Bradford protein assay (Bio-rad) and normalized to 1 µg/µL, followed by sample reduction and denaturation in 1x Laemmli sample buffer (Bio-rad) containing 2-mercaptoethanol. Protein lysates (10-15uL) were separated on Criterion TGX Tris-Glycine eXtended (TGX) precast gels (Bio-rad), transferred to PVDF membranes using the Trans-Blot Turbo system (Bio-rad), and incubated overnight with primary RPL5 (Abcam), α-Tubulin (Sigma Aldrich), Actin (Sigma Aldrich), phospho-CDC2/CDK1 (Tyr15), c-MYC, MDM2 or p53 (all from Cell Signaling) antibody, washed, and incubated for 1 hour with secondary Goat Anti-Mouse IgG-HRP or Goat Anti-Rabbit IgG-HRP antibodies (Thermo Fisher). Protein bands were visualized using chemiluminescent chemistry on an Azure C600 (Azure Biosystems). Quantification was performed using LI-COR Image Studio Lite software version 5.2.

### Xenografts in NOD-SCID/IL2γ^−/−^ (NSG) mice

Animal experiments were approved by the local ethics committee (P262-2015). NSG mice were recently purchased from Charles River laboratories and bred in our institute to obtain sufficient animals. 3*10^6^ breast cancer cells were injected subcutaneously in the left and right flank in a 1:1 mixture with Matrigel (Corning). Mice received fresh water containing 2 mg/mL doxycycline (Sigma Aldrich) and 2% sucrose (Sigma Aldrich) twice a week. The animals were monitored on a daily basis and sacrificed before tumors reached 2 cm^3^. MDA-MB-231 injected mice were sacrificed after 32 days and MCF7 injected mice were sacrificed after 58 days.

### Statistical analyses

All statistical analyses were performed using R and IBM SPSS 23 (IBM Analytics) softwares. For experimental work, a one-tailed *T*-test was used to determine whether *RPL5* knockdown increased breast cancer tumor weights and two-tailed paired Student's *t*-tests when comparing breast cancer cells in various assays.

## SUPPLEMENTARY MATERIALS FIGURES AND TABLES







## Abbreviations

T-ALL: T-cell leukemia
CLL: chronic lymphocytic leukemia
TCGA: The Cancer Genome Atlas
DBA: Diamond Blackfan Anemia
MDS: 5q- myelodysplastic syndrome
RMA: Robust Multi-Array Average
P: p-value
Q: p-value
FC: fold change
TP: primary tumor
TM: Metastatic Tumor
TB: Blood Tumor
BRCA: Breast invasive carcinoma
COADREAD: Colon-rectum adenocarcinoma
GBM: Glioblastoma multiforme
HNSC: Head and Neck squamous cell carcinoma
KIRC: Kidney renal clear cell carcinoma
KIRP: Kidney renal papillary cell carcinoma
LAML: Acute Myeloid Leukemia
LGG: Brain Lower Grade Glioma
LUAD: Lung adenocarcinoma
LUSC: Lung squamous cell carcinoma
OV: Ovarian serous cystadenocarcinoma
PRAD: Prostate adenocarcinoma
SKCM: Skin Cutaneous Melanoma
STAD: Stomach adenocarcinoma
THCA: Thyroid carcinoma
UCEC: Uterine Corpus Endometrial Carcinoma
rtTA3: reverse tetracycline-controlled trans-activator
NSG: NOD-SCID/IL2γ−/−
dox: doxycycline
ctrl: control
MSI: microsatellite instable
n.s.: non significant
Mbp: Megabase pair
UCEC: uterine corpus endometrial carcinoma
THCA: thyroid carcinoma
STAD: stomach adenocarcinoma
SKCM: skin cutaneous melanoma
PRAD: prostate adenocarcinoma
OV: ovarian serous cystadenocarcinoma
LUSC: lung squamous cell carcinoma
LUAD: lung adenocarcinoma
LGG: brain lower grade glioma
LAML: acute myeloid leukemia
KIRP: kidney renal papillary cell carcinoma
KIRC: kidney renal clear cell carcinoma
HNSC: head and neck squamous cell carcinoma
GBM: glioblastoma multiforme
COADREAD: colon-rectum adenocarcinoma
BRCA: breast invasive carcinoma
