# Supplementary material for: The ribosomal protein gene RPL5 is a haploinsufficient tumor suppressor in multiple cancer types
Source: Oncotarget. 2017 Jan 29;8(9):14462–78. doi: 10.18632/oncotarget.14895 (PMC5362418; doi:10.18632/oncotarget.14895)
Supplement: Supplementary file 1 [file oncotarget-08-14462-s001.pdf]

The ribosomal protein gene *RPL5* is a haploinsufficient tumor suppressor in multiple cancer types

Supplementary Material

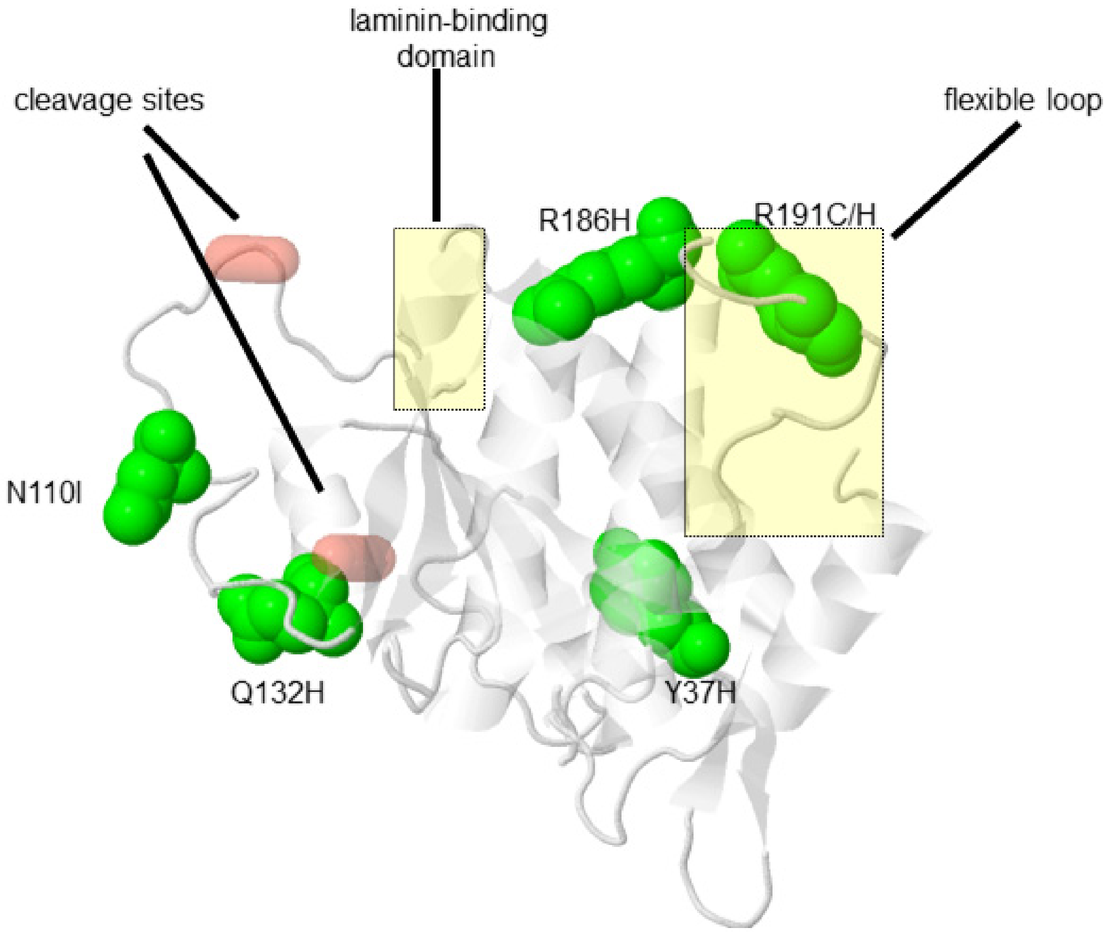

Supplementary Figure 1: Mapping of mutations affecting RPSA in STAD on the tridimensional protein structure. A significant cluster of mutations was detected in a flexible loop whose conformation regulates the accessibility of a laminin binding domain (aa 186-191, in green). The approximate localization of the laminin-binding domain and of the flexible loop regulating its accessibility is indicated by yellow boxes. Two additional mutations (N110I and Q132H, in green) are close to the cleavage sites (aa 116-117 and aa 136-137, in red) which regulate laminin-binding activity. The figure was generated using MuPIT Interactive (http://mupit.icm.jhu.edu/).

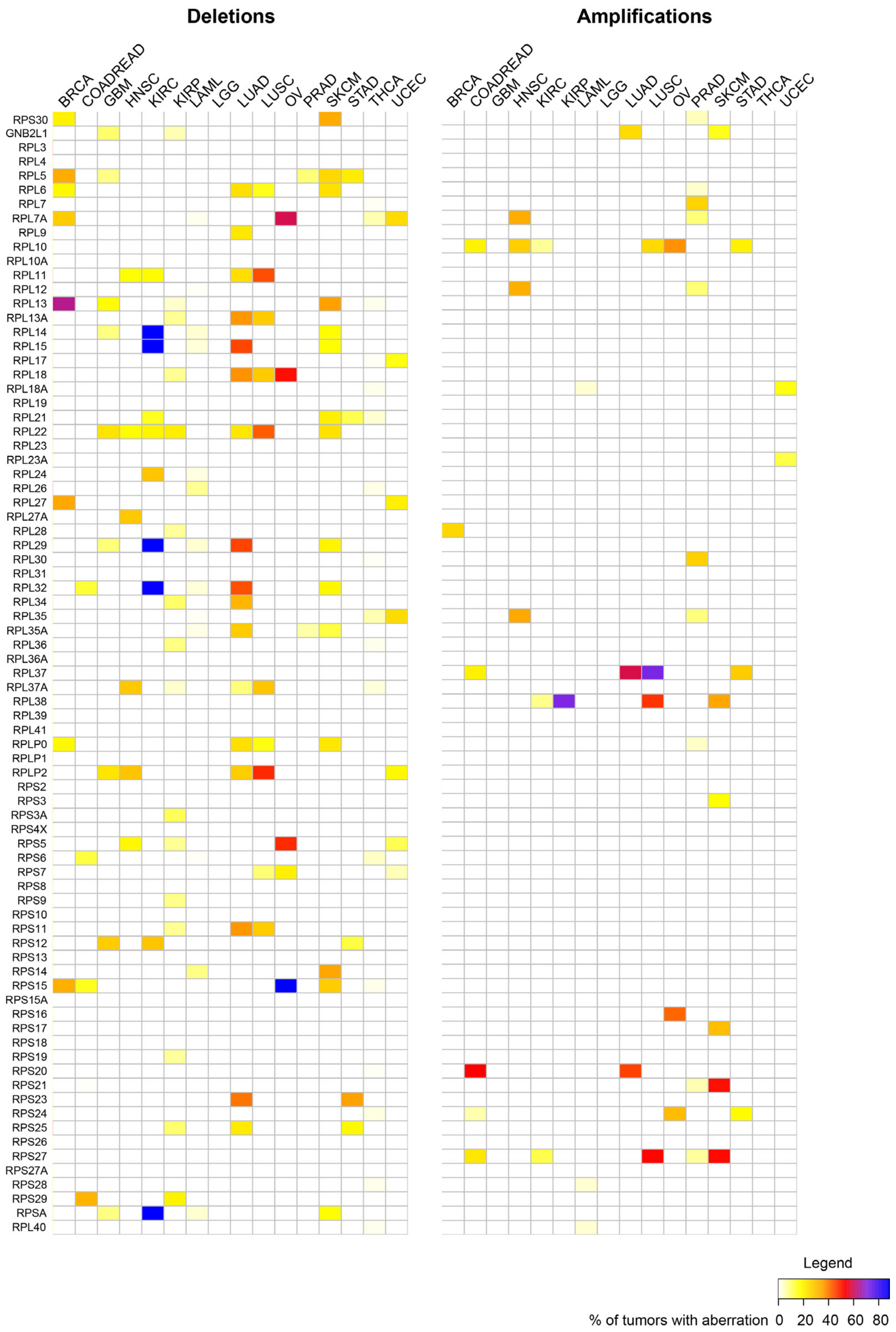

Supplementary Figure 2: Frequency of significant amplifications and deletions. Frequencies of significant deletions or amplifications as determined by Gistic 2.0 in ribosomal protein genes in the different cancer types.

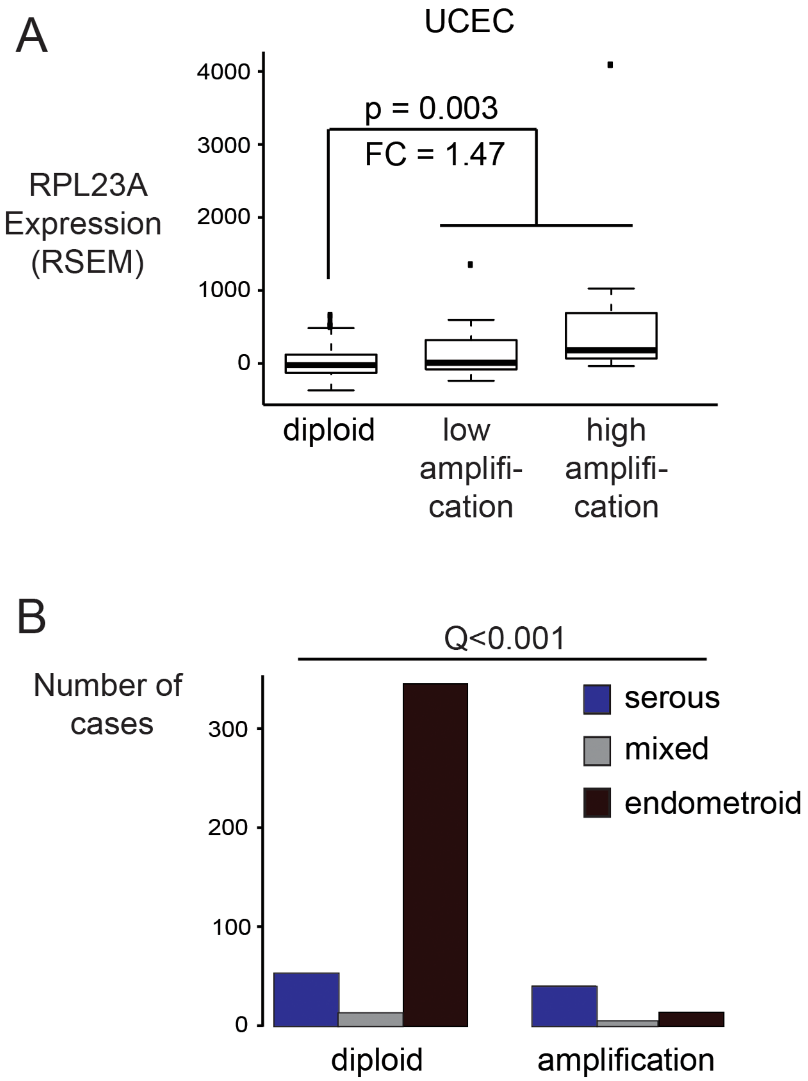

Supplementary Figure 3: RPL23A amplification in UCEC. A. Correlation between RPL23A copy number and mRNA expression levels (Illumina HiSeq, RSEM normalized) in UCEC. Expression values were centered around the diploid mean. The fold change (FC) between diploid and all amplifications (high and low) is reported, as well as the p-value (P) according to the Wilcoxon's test. **B**. Correlation between RPL23A amplification and UCEC histological subtype. Q: p-value from two-tailed Fisher's exact test corrected for multiple testing with the Benjamini and Hochberg method. Barplot and statistics were obtained from the Broad GDAC Firehose pipeline analysis "Correlation between copy number variation genes (focal events) and selected clinical features" (release 14/01/2015; doi:10.7908/C1M61HR5).

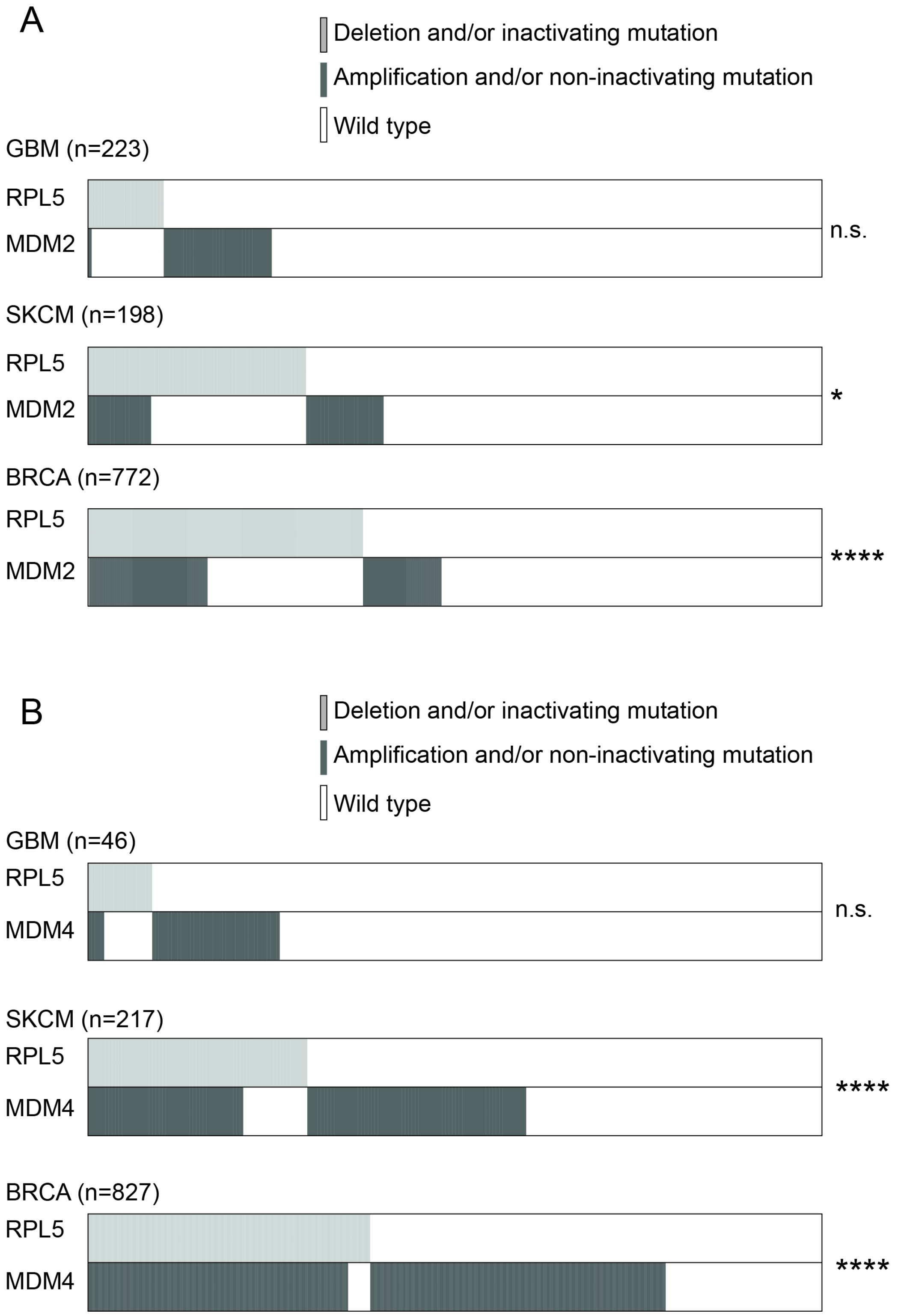

Supplementary Figure 4: Genetic interaction between RPL5 alterations and MDM2 and MDM4 alterations. **A**. Co-occurrence of *RPL5* and *MDM2* alterations in GBM, SKCM and BRCA. **B**. Co-occurrence of *RPL5* and *MDM4* alterations in GBM, SKCM and BRCA. *RPL5* defects: inactivating mutations or deletions; *MDM2* or *MDM4* alterations: non-inactivating mutations or amplifications.

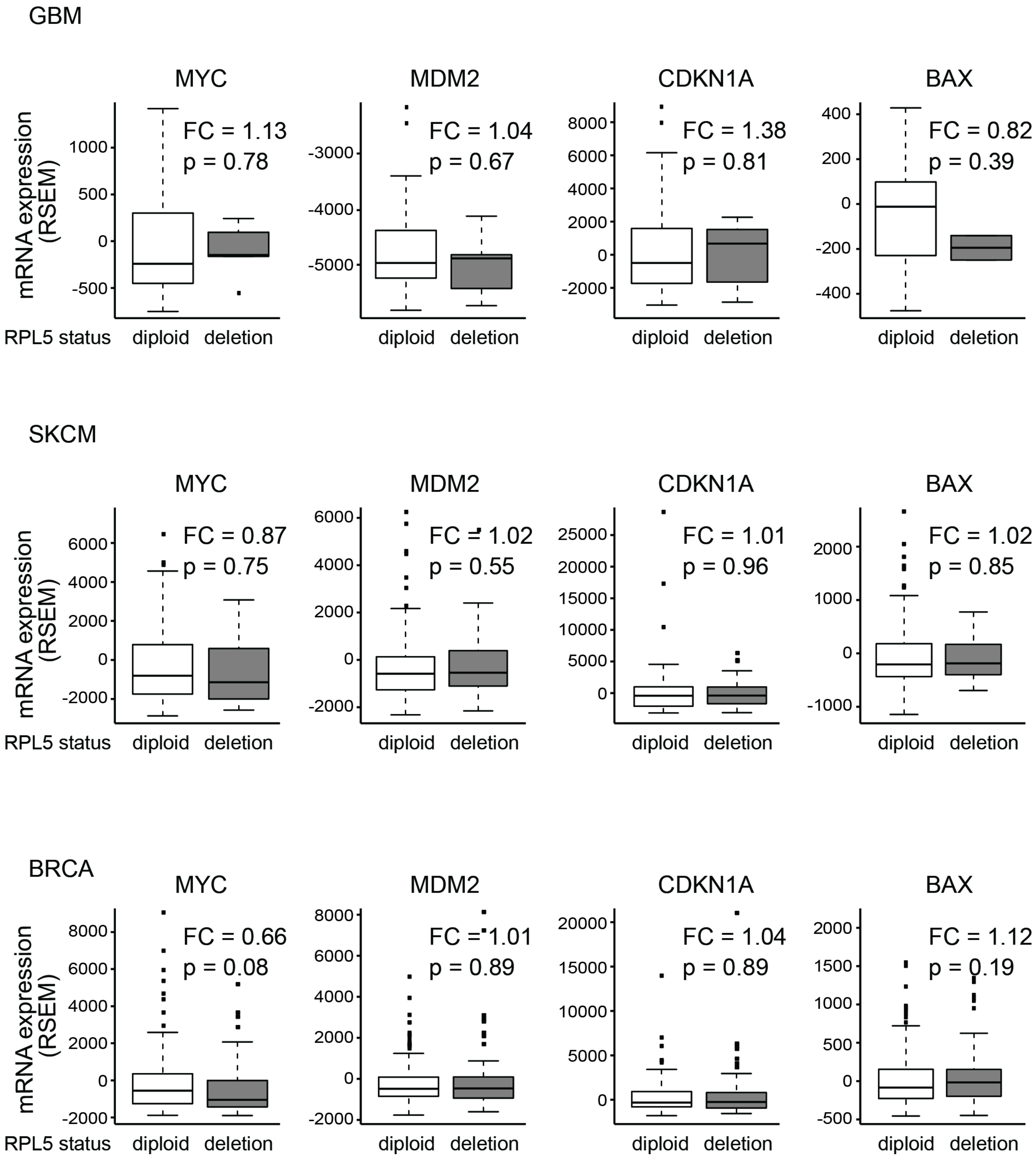

Supplementary Figure 5: mRNA expression levels of c-MYC and of the TP53 target genes MDM2, CDKN1A and BAX in GBM, SKCM and BRCA cases with diploid or heterozygously deleted copy number status for RPL5. P: p-value according to the Wilcoxon's test. FC: fold change (*RPL5* heterozygously deleted over *RPL5* diploid).

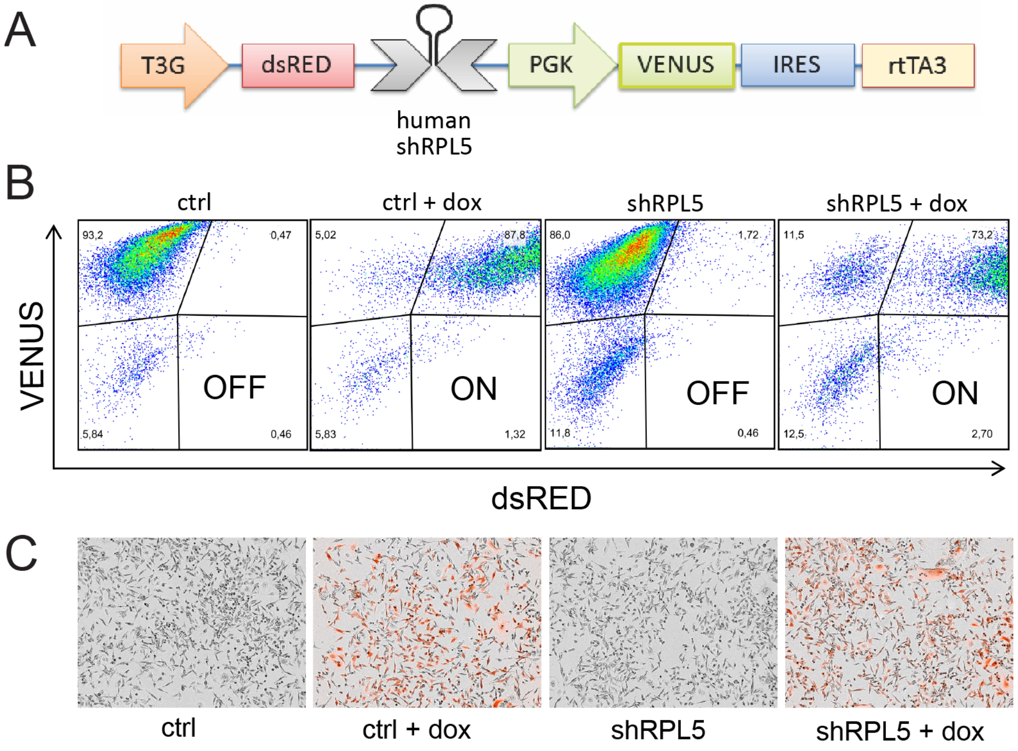

Supplementary Figure 6: Doxycycline inducible human RPL5 knockdown system. A. representation of the lentiviral inducible vector, which has constitutive VENUS expression driven by the PGK promoter and an rtTA3 (reverse tetracycline-controlled trans-activator) doxycycline responsive element that drives dsRED expression under the control of the TRE3G promoter as a measurement for RPL5 targeting shRNA transcription. **B**. Flow cytometric analysis of transduced cells with either the control vector or the shRNA targeting RPL5 with and without 2 ug/mL doxycycline. **C**. Microscopic pictures generated by the IncuCyte ZOOM System in phase-contrast and red fluorescence automated imaging mode of MDA-MB-231 cells, 72h after doxycycline administration.

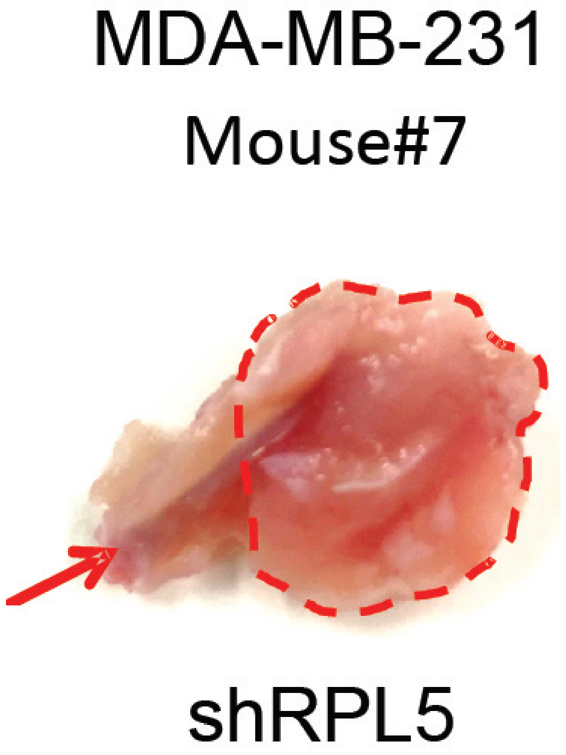

Supplementary Figure 7: Representative picture of an MDA-MB-231 shRPL5 tumor that encapsulated the bone.

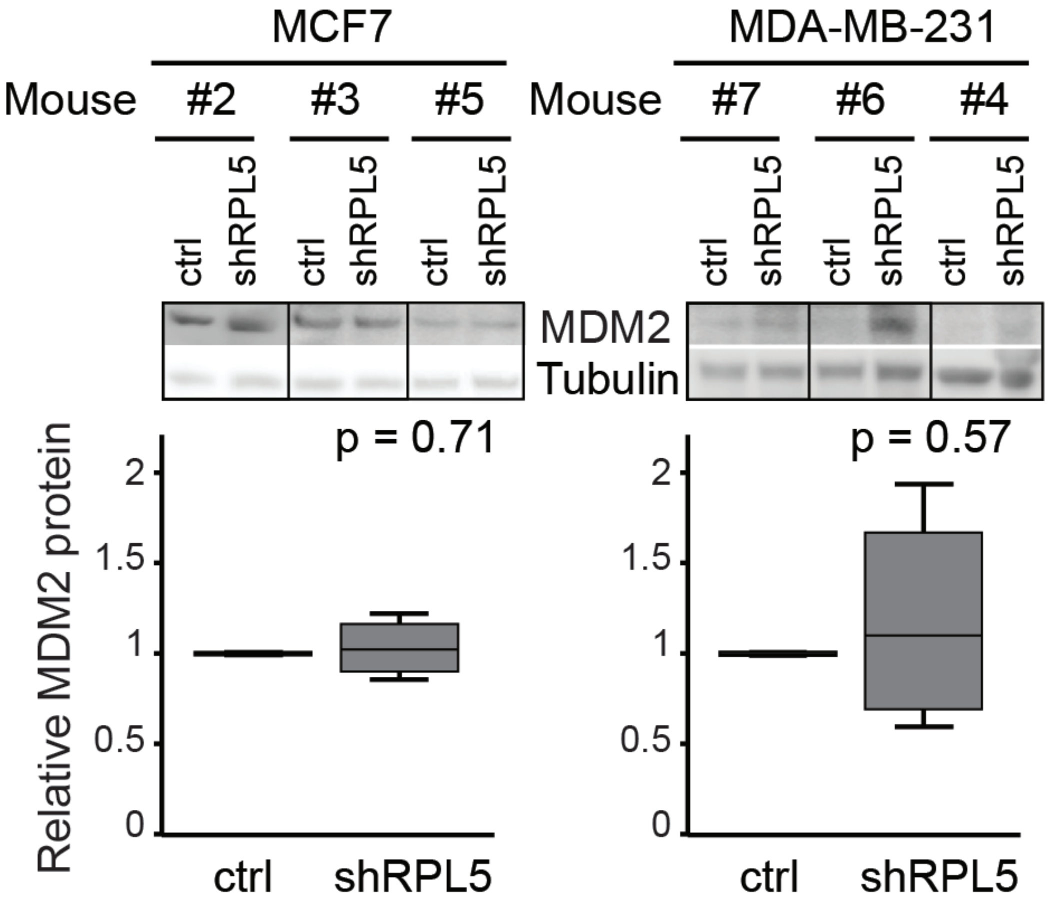

Supplementary Figure 8: Immunoblot analysis of MDM2 protein levels. Immunoblot analysis and the corresponding quantification of MCF (left) and MDA-MB-231 (right) tumors, comparing expression of the control and shRPL5 condition for MDM2 protein.

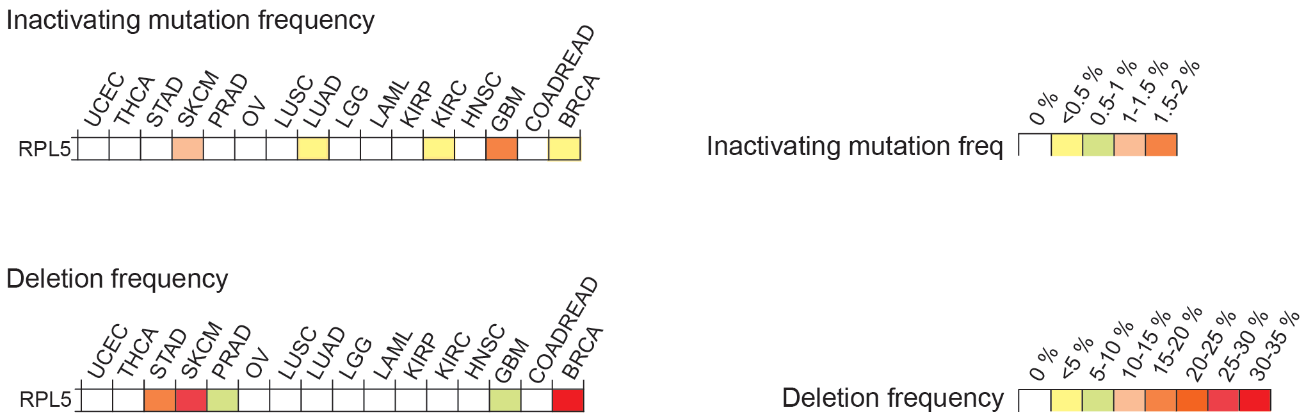

Supplementary Figure 9: All inactivating mutations and deletions in RPL5 in TCGA.

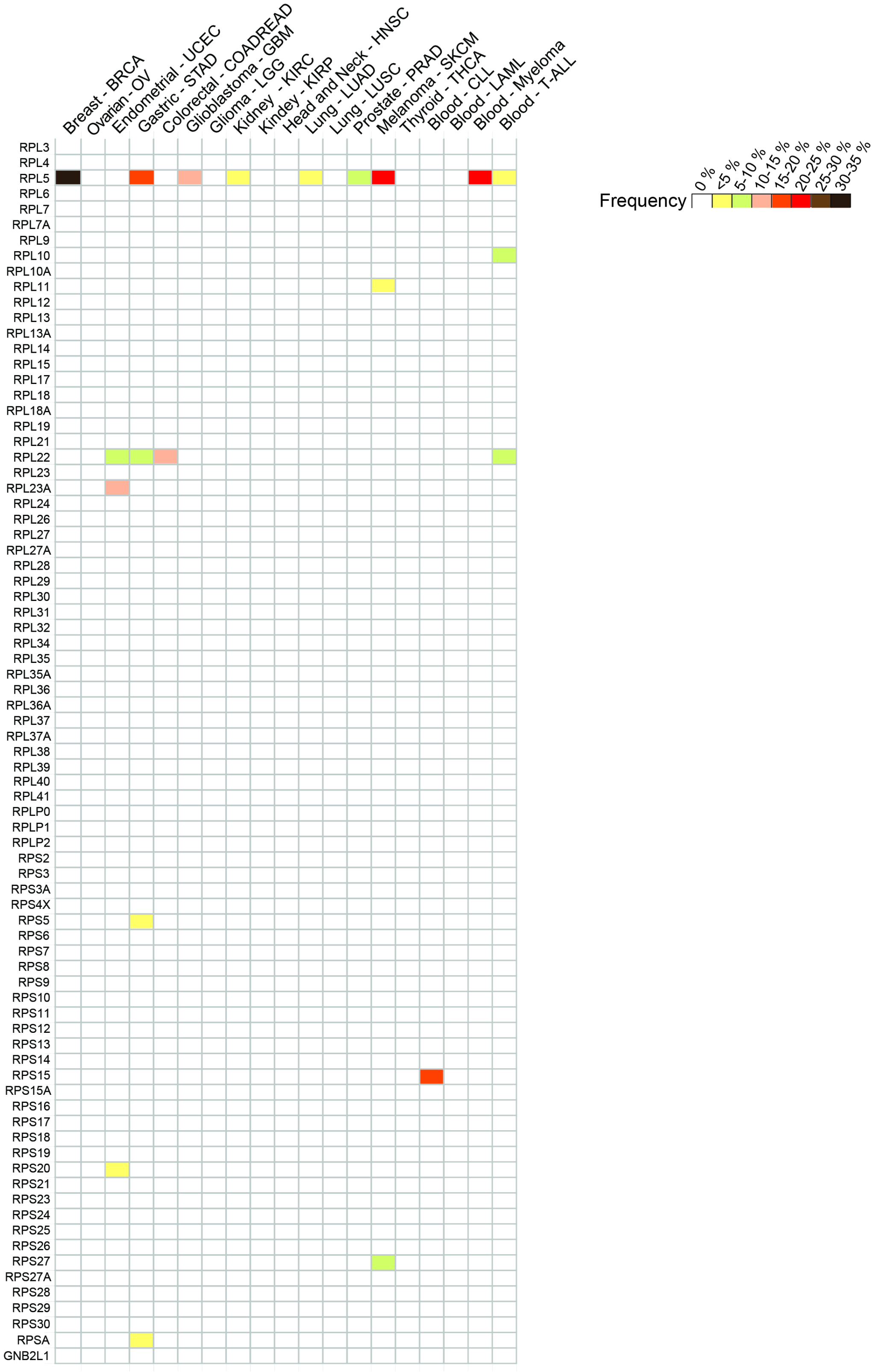

Supplementary Figure 10: Overview of all somatic ribosomal protein defects identified in this and other studies. Note that frequencies of RPL22 defects in endometrial, gastric and colorectal cancer have only been described in microsatellite instable (MSI) tumors. The frequency reported in this figure was obtained by multiplying the frequency of RPL22 defects in MSI tumors with the frequency of MSI in these tumor types.

Supplementary Table 1: Description of TCGA data. Number of samples for which somatic mutations, copy number changes, mRNA expression and clinical data are available for each cancer type. mRNA: expression data from Affymetrix HG-*U133A microarray platform*; mRNASeq: expression data from Illumina total RNA sequencing. TP: primary tumor; TM: Metastatic Tumor; TB: Blood Tumor.

			# analyzed cases				
**TCGA abbreviation**	**Cancer**	**Cohort**	**Somatic mutations**	**Copy Number**	**mRNA (array)**	**mRNASeq**	**Clinical data**
BRCA	Breast invasive carcinoma	TP	976	1016	-	1019	975
COADREAD	Colon-rectum adenocarcinoma	TP	223	589	-	574	602
GBM	Glioblastoma multiforme	TP	283	560	540	153	576
HNSC	Head and Neck squamous cell carcinoma	TP	306	452	-	424	379
KIRC	Kidney renal clear cell carcinoma	TP	417	504	-	506	505
KIRP	Kidney renal papillary cell carcinoma	TP	112	172	-	161	139
LAML	Acute Myeloid Leukemia	TB	197	197	-	179	200
LGG	Brain Lower Grade Glioma	TP	289	365	-	365	282
LUAD	Lung adenocarcinoma	TP	229	493	-	488	461
LUSC	Lung squamous cell carcinoma	TP	178	490	-	482	408
OV	Ovarian serous cystadenocarcinoma	TP	316	573	572	295	579
PRAD	Prostate adenocarcinoma	TP	251	278	-	278	186
SKCM	Skin Cutaneous Melanoma	TM	279	292	-	283	260
STAD	Stomach adenocarcinoma	TP	221	332	-	274	308
THCA	Thyroid carcinoma	TP	401	494	-	488	484
UCEC	Uterine Corpus Endometrial Carcinoma	TP	248	515	-	513	481

TP: Primary Tumor; TM: Metastatic Tumor; TB: Blood Tumor

Supplementary Table 2: Unfiltered mutations and copy number changes in all ribosomal protein genes. The number and percent of mutations for each ribosomal protein gene in each cancer type is provided together with the total number of samples for which mutation data is available. The frequency of amplification and deletion for each ribosomal protein gene in each cancer type is also provided together with the total number of samples for which copy number data is available. The significance of the copy number change according to Gistic 2.0 is indicated with '1' for significant and '0' for non-signficant.

Supplementary Table 3: Details on mutations in the six ribosomal protein genes identified as candidate cancer drivers. For each mutated residue, the aminoacid change and the type of mutation is reported, as well as the cancer type where the mutation was found. The impact of each mutation on interactions with other proteins or RNA, as predicted by the Mechismo tool, is also shown. TransFIC transformed functional impact scores are provided as estimates of the general functional impact of the mutations.

Supplementary Tables 2, 3 see in Supplementary Files
