# Supplementary material for: The ribosomal protein gene RPL5 is a haploinsufficient tumor suppressor in multiple cancer types
Source: Oncotarget. 2017 Jan 29;8(9):14462–78. doi: 10.18632/oncotarget.14895 (PMC5362418; doi:10.18632/oncotarget.14895)
Supplement: Supplementary file 3 [file oncotarget-08-14462-s003.docx]

| **Human name** | **Uniprot ID** | **Universal name** | **Mutated residues** | | | **Mechismo prediction of mutation impact on protein interactions** | | | | | **TransFIC transformed functional impact scores** | | |
| --- | --- | --- | --- | --- | --- | --- | --- | --- | --- | --- | --- | --- | --- |
|  |  |  | **Amino-acid change** | **Mutation** | **Cancer** | **interaction molecule** | **Effect type** | **Score** | **Confi**  **dence** | **Mechismo Score** | **SIFT** | **Polyphen2** | **Mutation Assessor** |
| RPS5 | P46782 | uS7 | A46S | missense | BRCA |  |  |  |  |  | medium | low | low |
|  |  |  | E3K | missense | BRCA |  |  |  |  |  | medium | low | low |
|  |  |  | E3Q | missense | BRCA |  |  |  |  |  | medium | low | low |
|  |  |  | E98K | missense | BRCA |  |  |  |  |  | high | medium | medium |
|  |  |  | F20L | missense | BRCA |  |  |  |  |  | high | medium | medium |
|  |  |  | T126A | missense | BRCA |  |  |  |  |  | high | low | medium |
|  |  |  | V113L | missense | BRCA |  |  |  |  |  | high | low | medium |
|  |  |  | V11G | missense | BRCA |  |  |  |  |  | low | low | low |
|  |  |  | S187F | missense | SKCM |  |  |  |  |  | medium | high | medium |
|  |  |  | A138V | missense | STAD |  |  |  |  |  | high | medium | medium |
|  |  |  | G80S | missense | STAD |  |  |  |  |  | medium | low | low |
|  |  |  | N203S | missense | STAD | RNA | disabling | -0.14 | high | 1.14 | high | medium | medium |
| RPS20 | P60866 | uS10 | R79C | missense | COADREAD, SKCM |  |  |  |  |  | medium | low | medium |
|  |  |  | E111K | missense | HNSC |  |  |  |  |  | medium | low | medium |
|  |  |  | P94A | missense | LUSC |  |  |  |  |  | medium | medium | medium |
|  |  |  | S31F | missense | SKCM |  |  |  |  |  | high | low | medium |
|  |  |  | E114K | missense | UCEC |  |  |  |  |  | medium | low | NA |
|  |  |  | H85R | missense | UCEC | RNA | enabling | -0.41 | high | 1.41 | high | medium | medium |
|  |  |  | R66I | missense | UCEC | RNA | disabling | -1.41 | high | 2.41 | high | medium | medium |
| RPSA | P08865 | uS2 | Q261R | missense | COADREAD |  |  |  |  |  | low | low | low |
|  |  |  | P105T | missense | LUAD |  |  |  |  |  | high | low | medium |
|  |  |  | E118K | missense | SKCM |  |  |  |  |  | high | high | low |
|  |  |  | E196K | missense | SKCM |  |  |  |  |  | high | low | low |
|  |  |  | E211K | missense | SKCM |  |  |  |  |  | low | medium | low |
|  |  |  | L154R | missense | SKCM |  |  |  |  |  | high | high | medium |
|  |  |  | P199L | missense | SKCM |  |  |  |  |  | medium | high | low |
|  |  |  | S152F | missense | SKCM |  |  |  |  |  | high | medium | medium |
|  |  |  | S190F | missense | SKCM |  |  |  |  |  | medium | medium | medium |
|  |  |  | T112S | missense | SKCM |  |  |  |  |  |  |  |  |
|  |  |  | W117* | nonsense | SKCM |  |  |  |  |  |  |  |  |
|  |  |  | N110I | missense | STAD | RNA | disabling | -0.78 | high | 1.78 | high | low | medium |
|  |  |  | Q132H | missense | STAD | RNA | enabling | 0.28 | high | 1.28 | high | medium | medium |
|  |  |  | R186H | missense | STAD |  |  |  |  |  | medium | low | low |
|  |  |  | R191C | missense | STAD |  |  |  |  |  | medium | low | low |
|  |  |  | R191H | missense | STAD |  |  |  |  |  | medium | low | low |
|  |  |  | Y37H | missense | STAD |  |  |  |  |  | high | low | low |
|  |  |  | E225D | missense | UCEC |  |  |  |  |  | low | medium | medium |
| RPL11 | P62913 | uL5 | F166L | missense | COADREAD |  |  |  |  |  | low | low | low |
|  |  |  | A2_splice | splice | GBM |  |  |  |  |  |  |  |  |
|  |  |  | G30fs | fs | GBM |  |  |  |  |  |  |  |  |
|  |  |  | I151F | missense | KIRC |  |  |  |  |  | high | low | medium |
|  |  |  | F107V | missense | KIRP |  |  |  |  |  | high | medium | medium |
|  |  |  | V89_splice | splice | KIRP |  |  |  |  |  |  |  |  |
|  |  |  | S100L | missense | LUAD |  |  |  |  |  | high | low | medium |
|  |  |  | E7fs | fs | PRAD |  |  |  |  |  |  |  |  |
|  |  |  | L15F | missense | PRAD |  |  |  |  |  | medium | medium | medium |
|  |  |  | K118T | missense | SKCM | RNA | disabling | -0.46 | high | 1.46 | high | medium | medium |
|  |  |  | L33M | missense | SKCM |  |  |  |  |  | high | high | medium |
|  |  |  | Q167* | nonsense | SKCM |  |  |  |  |  |  |  |  |
|  |  |  | S59F | missense | SKCM | RNA | disabling | -0.26 | high | 1.26 | high | medium | medium |
|  |  |  | A142T | missense | UCEC |  |  |  |  |  | low | low | low |
|  |  |  | R13W | missense | UCEC |  |  |  |  |  | medium | low | medium |
|  |  |  | R146H | missense | UCEC |  |  |  |  |  | low | low | low |
| RPL23A | P62750 | uL23 | L147F | missense | LUAD |  |  |  |  |  | medium | medium | medium |
|  |  |  | Y144D | missense | LUAD |  |  |  |  |  | high | medium | low |
|  |  |  | E133D | missense | OV |  |  |  |  |  | low | medium | low |
|  |  |  | K39del | fs | STAD |  |  |  |  |  |  |  |  |
|  |  |  | A75D | missense | UCEC | RNA | disabling | -0.15 | high | 1.15 | low | medium | low |
|  |  |  | I40T | missense | UCEC |  |  |  |  |  | medium | low | medium |
|  |  |  | K39in_frame_del | indel | UCEC |  |  |  |  |  |  |  |  |
|  |  |  | P18T | missense | UCEC |  |  |  |  |  | medium | low | medium |
|  |  |  | R41C | missense | UCEC | RNA | disabling | -1.67 | medium | 2.67 | high | low | low |
| RPL5 | P46777 | uL18 | A97G | missense | BRCA |  |  |  |  |  | high | low | medium |
|  |  |  | N57fs | fs | BRCA |  |  |  |  |  |  |  |  |
|  |  |  | K258N | missense | COADREAD, UCEC |  |  |  |  |  | medium | low | medium |
|  |  |  | G71E | missense | GBM | RNA | enabling | 3.16 | medium | 4.16 | high | high | medium |
|  |  |  | K5fs | fs | GBM |  |  |  |  |  |  |  |  |
|  |  |  | L109_splice | splice | GBM |  |  |  |  |  |  |  |  |
|  |  |  | Q63_splice | splice | GBM | RNA | unknown | 0 | medium | 1 |  |  |  |
|  |  |  | V159fs | fs | GBM | RNA | unknown | 0 | medium | 1 |  |  |  |
|  |  |  | Y16* | nonsense | GBM | RNA | unknown | 0 | medium | 1 |  |  |  |
|  |  |  | Y226H | missense | GBM | RNA | enabling | 0.16 | medium | 1.16 | high | medium | medium |
|  |  |  | R265_splice | splice | KIRC |  |  |  |  |  |  |  |  |
|  |  |  | Y44* | nonsense | KIRC |  |  |  |  |  |  |  |  |
|  |  |  | Y45del | indel | KIRC |  |  |  |  |  |  |  |  |
|  |  |  | E238* | nonsense | LUAD |  |  |  |  |  |  |  |  |
|  |  |  | P181T | missense | LUSC |  |  |  |  |  | medium | medium | medium |
|  |  |  | T154I | missense | LUSC |  |  |  |  |  | high | low | medium |
|  |  |  | N94D | missense | PRAD |  |  |  |  |  | high | low | medium |
|  |  |  | F113fs | fs | SKCM | RNA | unknown | 0 | medium | 1 |  |  |  |
|  |  |  | F3L | missense | SKCM |  |  |  |  |  | medium | low | low |
|  |  |  | G169V | missense | SKCM |  |  |  |  |  | high | medium | medium |
|  |  |  | G91N | missense | SKCM |  |  |  |  |  |  |  |  |
|  |  |  | K5* | nonsense | SKCM |  |  |  |  |  |  |  |  |
|  |  |  | P47fs | fs | SKCM |  |  |  |  |  |  |  |  |
|  |  |  | S176_splice | splice | SKCM | RNA | unknown | 0 | medium | 1 |  |  |  |
|  |  |  | M208T | missense | STAD |  |  |  |  |  | high | low | medium |
|  |  |  | A205V | missense | UCEC |  |  |  |  |  | medium | low | medium |
|  |  |  | E82K | missense | UCEC |  |  |  |  |  | high | low | medium |
|  |  |  | R209H | missense | UCEC |  |  |  |  |  | medium | low | low |
|  |  |  | R248Q | missense | UCEC |  |  |  |  |  | high | medium | low |
|  |  |  | S185F | missense | UCEC |  |  |  |  |  | medium | low | low |
|  |  |  | V231I | missense | UCEC |  |  |  |  |  | low | low | low |
